# Improving tumor budding reporting in colorectal cancer: a Delphi consensus study

**DOI:** 10.1007/s00428-021-03059-9

**Published:** 2021-03-01

**Authors:** Tariq Sami Haddad, Alessandro Lugli, Susan Aherne, Valeria Barresi, Benoît Terris, John-Melle Bokhorst, Scarlet Fiona Brockmoeller, Miriam Cuatrecasas, Femke Simmer, Hala El-Zimaity, Jean-François Fléjou, David Gibbons, Gieri Cathomas, Richard Kirsch, Tine Plato Kuhlmann, Cord Langner, Maurice B. Loughrey, Robert Riddell, Ari Ristimäki, Sanjay Kakar, Kieran Sheahan, Darren Treanor, Jeroen van der Laak, Michael Vieth, Inti Zlobec, Iris D. Nagtegaal

**Affiliations:** 1 Department of Pathology, Radboud University Medical Center, P.O. Box 9101, 6525 GA Nijmegen, Netherlands; 2 University of Bern, Bern, Switzerland; 3 St. Vincent’s University Hospital, Dublin, Ireland; 4 University College Dublin, Dublin, Ireland; 5 University of Verona, Verona, Italy; 6 Cochin Hospital, Paris, France; 7 University of Paris, Paris, France; 8 University of Leeds, Leeds, England; 9 Hospital Clínic, Barcelona, Spain; 10 Dynacare Laboratories, Brampton, Canada; 11 Saint-Antoine Hospital, Paris, France; 12 Cantonal Hospital Baselland, Liestal, Switzerland; 13 Mt. Sinai Hospital, Toronto, Canada; 14 Herlev Hospital, Copenhagen, Denmark; 15 Medical University of Graz, Graz, Austria; 16 Royal Victoria Hospital and Queen’s University, Belfast, United Kingdom; 17 University of Helsinki, Helsinki, Finland; 18 HUSLAB, Helsinki University Hospital, Helsinki, Finland; 19 University of California, San Francisco, San Francisco, CA USA; 20 Linköping University, Linköping, Sweden; 21 University of Bayreuth, Bayreuth, Germany

**Keywords:** Colorectal cancer, Tumor budding, Delphi study, ITBCC

## Abstract

Tumor budding is a long-established independent adverse prognostic marker in colorectal cancer, yet methods for its assessment have varied widely. In an effort to standardize its reporting, a group of experts met in Bern, Switzerland, in 2016 to reach consensus on a single, international, evidence-based method for tumor budding assessment and reporting (International Tumor Budding Consensus Conference [ITBCC]). Tumor budding assessment using the ITBCC criteria has been validated in large cohorts of cancer patients and incorporated into several international colorectal cancer pathology and clinical guidelines. With the wider reporting of tumor budding, new issues have emerged that require further clarification. To better inform researchers and health-care professionals on these issues, an international group of experts in gastrointestinal pathology participated in a modified Delphi process to generate consensus and highlight areas requiring further research. This effort serves to re-affirm the importance of tumor budding in colorectal cancer and support its continued use in routine clinical practice.

**Supplementary Information:**

The online version contains supplementary material available at 10.1007/s00428-021-03059-9.

## Introduction

The phenomenon of tumor budding (TB), defined as single cells and isolated cells clusters up to 4 cells at the tumor invasive front, has captured the interest of pathologists, clinicians, and researchers since it was first described in the 1950s [1]. A large body of evidence has since firmly established TB as a strong and independent predictor of lymph node metastasis (LNM), disease recurrence, and cancer-related death in patients with colorectal cancer (CRC) [2]. Despite wide variation in the criteria, methods, and reporting systems for the assessment of TB across different studies, TB has proved a remarkably consistent predictor of adverse outcome in CRC. However, until recently, the absence of a standardized scoring system made it difficult to implement TB in routine pathology practice. This prompted a group of international experts to meet in Bern, Switzerland, in 2016 to host the International Tumor Budding Consensus Conference (ITBCC) to reach consensus on a single, evidence-based method for TB assessment and reporting in CRC [3]. Since the publication of the ITBCC consensus recommendations in 2017, TB has been incorporated as an additional prognostic factor in the World Health Organization Classification of Tumors (2019), the Tumor, Nodes, Metastasis (TNM) staging system, and included in the reporting guidelines and protocols of the College of American Pathologists (CAP) [4], the National Comprehensive Cancer Network (NCCN) [5], and the International Collaboration on Cancer Reporting [6]. The ITBCC recommendations have since been validated in several large cohorts of colorectal cancer and a prospective multi-center clinical trial [7–12]. With the wider application of TB in both the research setting and clinical practice, several issues have emerged which require further clarification. Some relate to aspects of TB assessment, risk stratification based on TB in different clinical scenarios, and the relationship of TB to other biomarkers at the invasive front. Since the ITBCC recommendations were not intended to be an end point, but rather a foundation for further research, refinement, and periodic review, its members organized a modified Delphi consensus process. The aim of this effort was to reach consensus on a number of emerging issues, ongoing challenges, and areas in need of further research.

## Material and methods

**Fig. 1:**
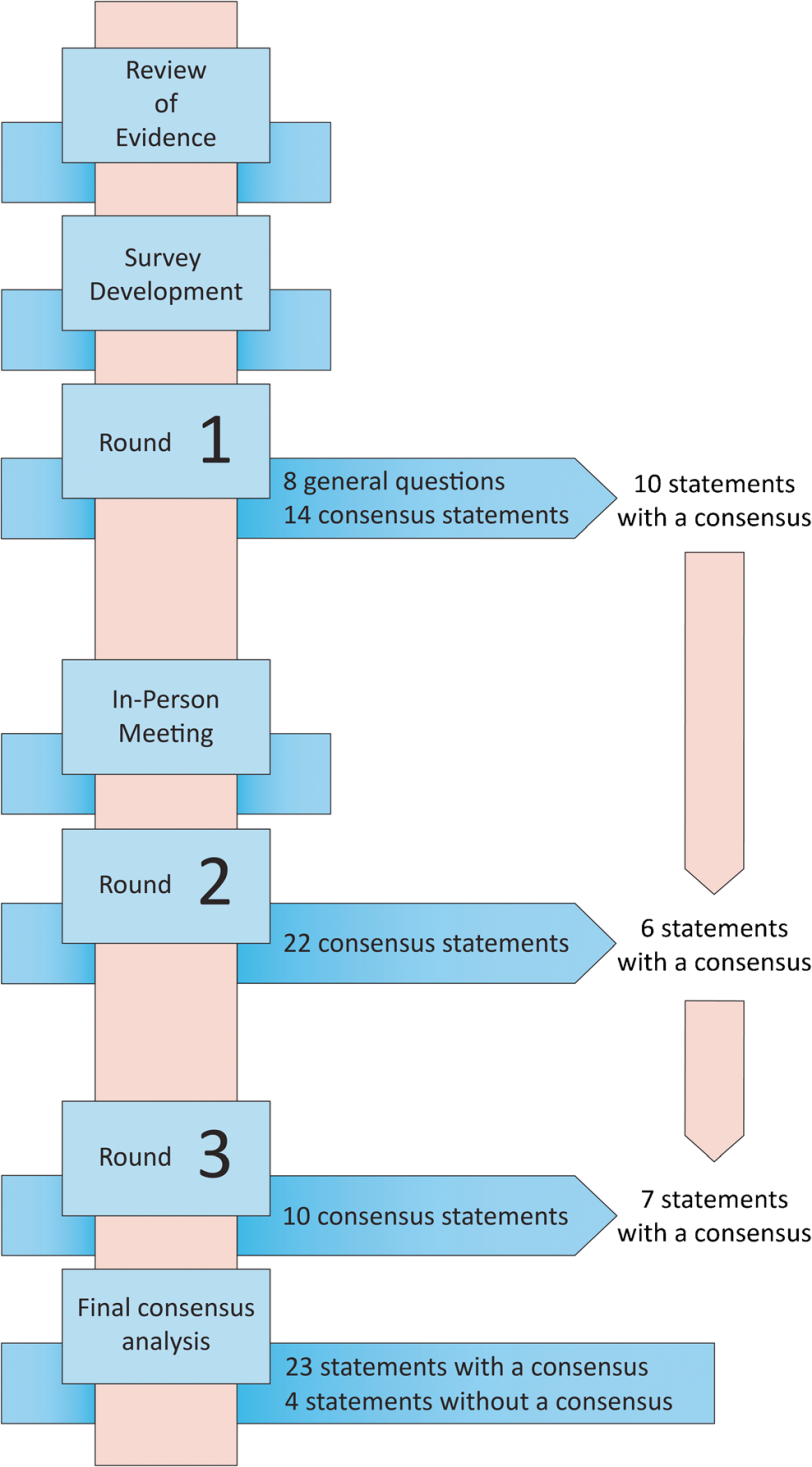
Modified Delphi consensus format. The survey consisted of 3 rounds and an in-person meeting. A total of 23 statements achieved a consensus and 4 statements did not

A group of 27 international pathologists with expertise in colorectal cancer pathology were invited to participate in the Delphi consensus survey. A total of 14 experts agreed to participate. The format of the consensus process is outlined in Fig. 1. The survey implemented an adapted version of the original Delphi method [13], which is designed to achieve consensus among a group of experts using a series of surveys. Based on a review of the TB literature (Supplementary Data Figure 1), two independent non-voting moderators (I.N and A.L) generated the Round 1 questionnaire consisting of a series of general questions and consensus statements. Rather than using open-ended questions in the first round, as has been done in other studies, general questions and agree/disagree statements were used. The general questions related to the implementation of TB by pathologists, the awareness of TB among clinicians, and the application of TB in clinical decision-making at the experts’ institutions.

The consensus statements solicited the experts’ opinions regarding the assessment and reporting of TB, its clinical application (particularly in malignant colorectal polyps (pT1) and stage II CRC), and its relationship to other biomarkers at the invasive front. The moderators disseminated the questionnaire to the participants using SurveyMonkey on August 7, 2019. Participants were able to provide commentary on questions to support their responses or express a particular opinion. Participants were also able to state “no opinion” on consensus statements. Individual votes were anonymized, and a consensus was considered achieved when ≥70% of participants either agreed/strongly agreed or disagreed/strongly disagreed with the statement. After the results of Round 1 were gathered, the moderators reviewed the results. Statements which did not achieve consensus (Supplementary Data Table 1) were later re-formulated and used by the moderators to generate new consensus statements for the next round of questioning.

An in-person meeting with the participants of the survey took place in Nice, France, on September 10, 2019. Prior to the meeting, an e-book containing all relevant TB studies published since ITBCC 2016 (Supplementary Data Figure 1) was circulated to the participants to ensure that all were familiar with new evidence related to TB in CRC. Participants were asked to review the e-book prior to the meeting. At the meeting, the results of the first round of the survey were presented by the moderators, and all questions and statements were then opened to discussion and debate among participants. Opinions expressed during this discussion as well as the statements not achieving consensus (Supplementary Data Table 1) were later used by the moderators to formulate new consensus statements for the next round of questioning. The moderators generated the statements for Round 2 and disseminated them on December 19, 2019. The statements which did not achieve consensus (Supplementary Data Table 2) and corresponding commentary for Round 2 were used to generate new consensus statements for the next round. This yielded a final series of 10 consensus statements for Round 3 which were sent out on February 3, 2020. A final consensus analysis was conducted and incorporated all consensus statements achieving consensus as well as statements which could not achieve consensus by Round 3 (11 from Round 1, 6 from Round 2, and 10 from Round 3).

## Results

All 14 experts who agreed to participate in the study completed all three rounds of the Delphi consensus process. The results are depicted in a flowchart outlining the survey process (Fig. 1). A total of 23 statements reached a consensus, while 4 did not.

### General

**Fig. 2:**
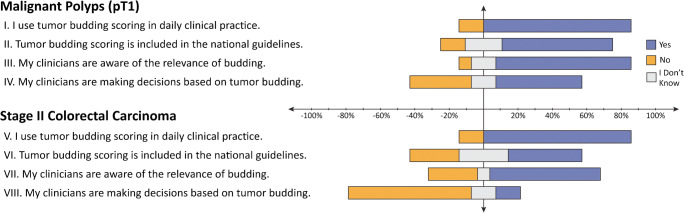
General questions. Responses to questions regarding the usage and awareness of tumor budding in the experts’ clinical setting

In the first round, participants received a series of 8 general questions regarding the implementation, clinician awareness, and clinical use of TB in their clinical practice (Fig. 2). Eighty-five percent of participants indicated that they routinely report TB in both pT1 and stage II CRC, while 64% and 43% indicated that scoring of TB was included in their national guidelines for pT1 and stage II CRC, respectively (I, V, II, VI). Seventy-nine and 64 percent indicated that their clinicians are aware of the relevance of TB in pT1 and stage II CRCs, respectively (III, VII), while 50% and 15% indicated that TB is taken into account in clinical decision-making for pT1 and stage II CRCs, respectively (IV, VIII).

### Consensus statements

**Fig. 3:**
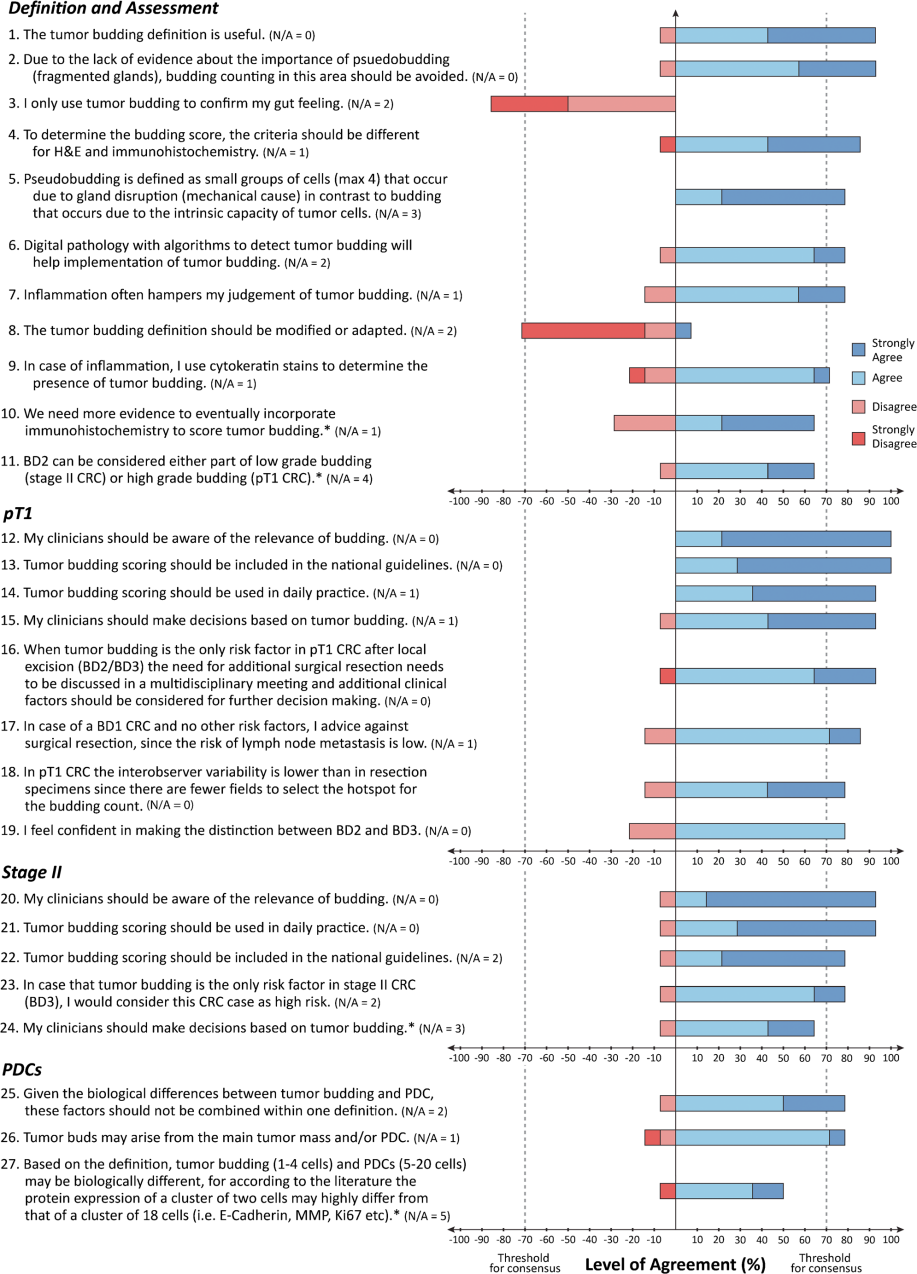
Consensus statements. Results of Delphi study after final consensus analysis. Each statement has a corresponding bar graph where the level of agreement/disagreement (%) is depicted. Statements are ranked within each subcategory from greatest to least degree of consensus. No opinion (N/A) votes are votes considered neither agree nor disagree for all corresponding consensus statements. All experts were able to vote “no opinion” on each statement. *Statements which did not achieve consensus

The final consensus statements are listed graphically to depict consensus and levels of agreement among experts with corresponding no opinion votes per statement (Fig. 3).

#### Definition and assessment

**Fig. 4:**
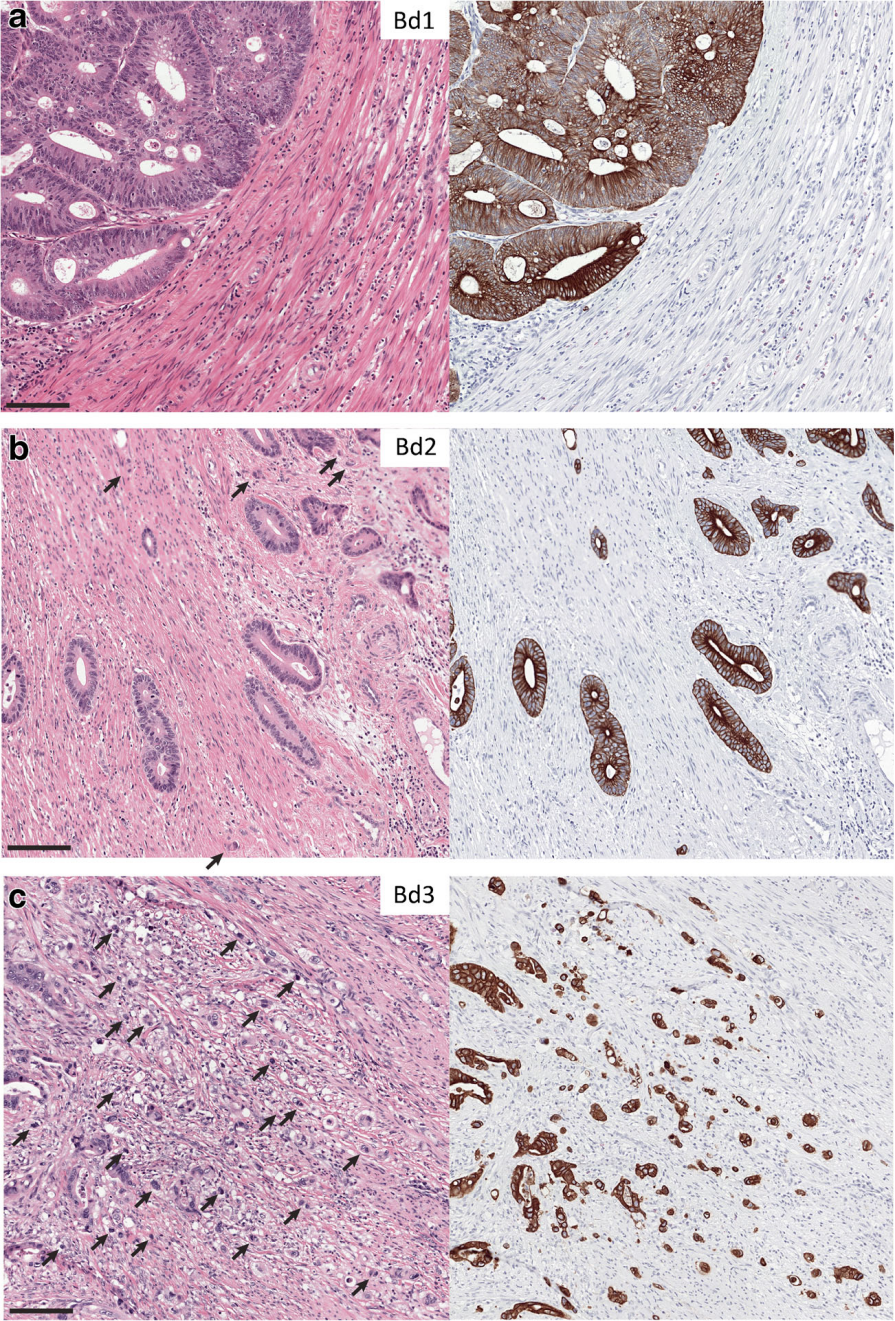
Tumor budding scores. Examples of different tumor budding scores (hotspot, 0.785 mm^2^) at the invasive front of colorectal cancer based on the ITBCC 2016. **a** Bd1 (low), **b** Bd2 (intermediate), **c** Bd3 (high). Each case was re-stained with pan-cytokeratin (AE1/AE3), and the same region is depicted on the right. Arrows indicate tumor budding. Scale bar = 125 μm

There was consensus that the definition of TB and its method of assessment and scoring as recommended by the ITBCC remains useful and should be retained and that there was no new evidence to support modifying this definition (#1, #8, Box 1, Fig. 4). There is also an agreement that using digital pathology algorithms may help with the implementation of tumor budding within the clinical workflow (#6).
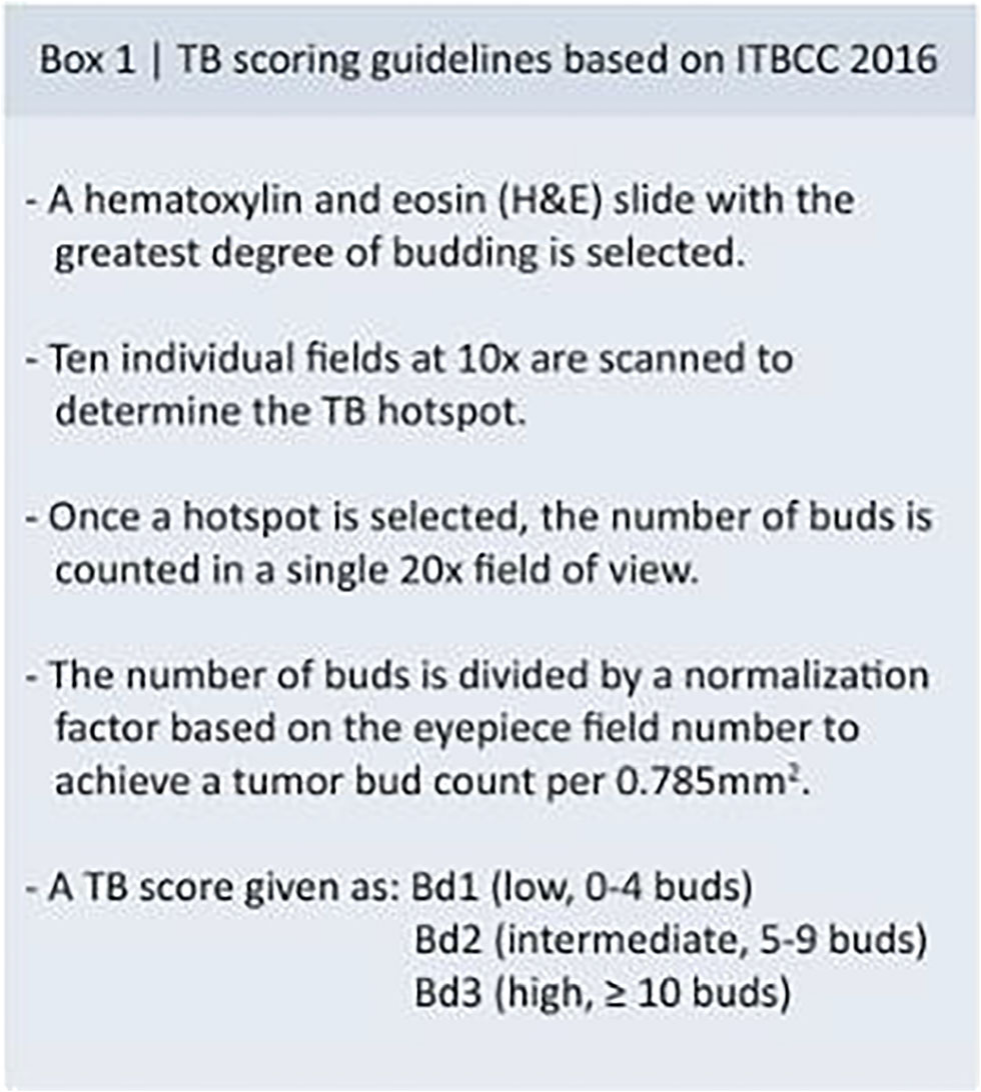

**Fig. 5:**
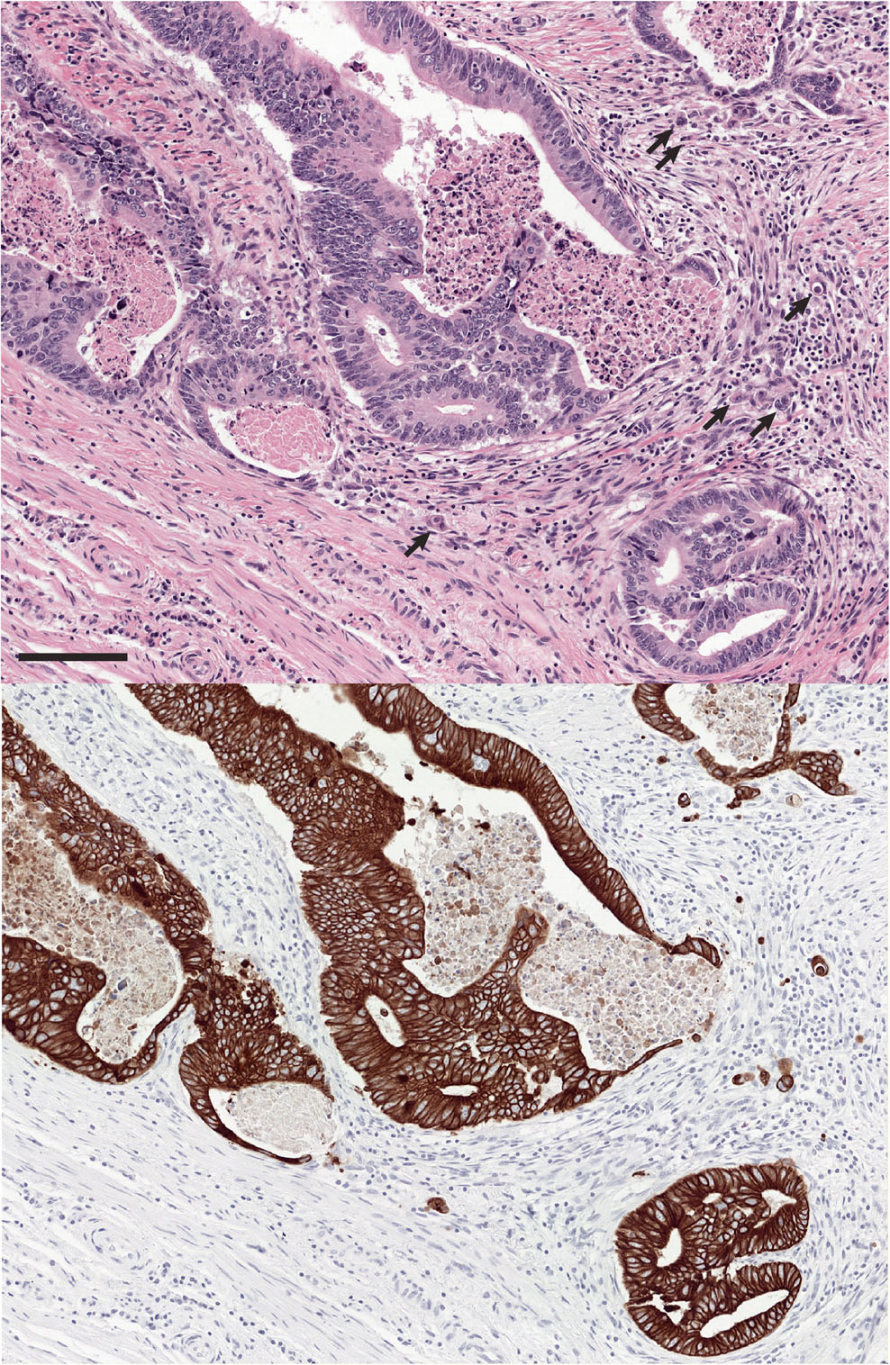
Pseudobudding. Example of a region (0.785 mm^2^) at the invasive margin with gland rupture and suspected pseudobudding. The slide was re-stained with pan-cytokeratin (AE1/AE3), and the same region is depicted on the right. Arrows indicate pseudobudding. Scale bar = 125 μm

Several questions addressed challenges related to TB assessment in areas with substantial inflammation which can result in tumor fragmentation mimicking TB or can obscure true TB. Ninety three percent of participants agreed that TB counting should be avoided in areas of tumor/glandular fragmentation caused by heavy inflammation (#2). Seventy-nine percent supported the term “pseudobudding” (defined as individual cells or small groups of cells resulting from fragmentation of glands secondary to inflammation) to describe this phenomenon, which likely differs biologically from TB (#5) (Fig. 5). Seventy-nine percent indicated that inflammation often hinders their assessment of TB on H&E (#7), while 71% indicated that they use pan-cytokeratin immunohistochemistry (IHC) in this setting to better visualize TB (#9), with final bud counting performed on H&E.

#### Clinical scenarios

1. Malignant colorectal polyps (pT1)

With regard to TB in pT1 CRC, there was unanimous (100%) agreement that clinicians should be aware of its relevance and that TB should be included in national guidelines (#12, #13). Ninety-three percent felt that TB should be routinely scored and that clinicians should take TB into account in clinical decision-making after local resection of pT1 CRC (#14, #15). If TB (Bd2/Bd3) was the only risk factor present, the need for additional surgical resection should be discussed in a multidisciplinary meeting in which additional clinical factors should also be considered (#16). Experts agreed that when low TB (Bd1) is observed in pT1 CRC cases with no other risk factors present, surgical resection is not advised as the risk of LNM is considered very low (#17).

(2) Stage II colorectal carcinoma

With regard to TB in stage II CRC, there was strong consensus that TB should be routinely scored and that clinicians should be aware of its relevance (93% agreement) (#21, #20), while 79% agreed that TB should be included in national guidelines (#22). Seventy-nine percent of participants also agreed that if TB were the only risk factor in stage II CRC, then this should be considered a high-risk stage II CRC (#23). Participants did not reach consensus on whether clinicians should make decisions based solely on TB in stage II CRC (#24).

#### Poorly differentiated clusters (PDCs)

PDCs are clusters of cancer cells in the stroma composed of five or more cells and which lack a glandular structure [14]. There was consensus that TB and PDC are different and should not be combined within one definition (in keeping with ITBCC recommendations) (#25). There was also consensus that TB may arise from the main tumor mass and/or PDCs (#26).

## Discussion

This survey, using a modified Delphi process and a panel of 14 experts in gastrointestinal pathology, was undertaken to evaluate new evidence related to TB, establish consensus on best practice, and identify areas in need of future research. The survey effectively generated consensus on several aspects of TB, the most important of which are discussed below.

There was strong consensus that TB scoring based on the ITBCC recommendations remains effective and should be used in daily practice for malignant colorectal polyps (pT1) and stage II CRC. This was underpinned by several large published studies validating the ITBCC recommendations [7–12] and the absence of any new evidence suggesting the need for their modification.

There was consensus that heavy inflammation at the invasive front often poses challenges in TB assessment. These challenges may be a consequence of tumor fragmentation by inflammatory cells resulting in detached tumor cells that may be mistaken for TB (“pseudobudding”) or due to inflammatory cells obscuring or mimicking TB. There was agreement that the term “pseudobudding” should apply to small groups of cells (maximum 4) that occur due to external influences such as inflammation and mechanical causes such as cutting artifacts. From a practical perspective, true tumor buds infiltrate the peritumoral stroma, while pseudobuds are typically surrounded by a mix of inflammatory cells, lack overt stromal infiltration, and tend to be limited to the immediate vicinity of fragmented glands/tumor nests (often following their original contours). Their biology likely differs from that of true TB since they are assumed to result from a reactive rather than active process; moreover, the heavy inflammation that produces pseudobudding is a feature generally associated with favorable outcomes. For these reasons, there was broad consensus that TB counting should be avoided in areas showing pseudobudding. Further studies need to be conducted to provide molecular evidence for the distinction between tumor budding and psuedobudding.

It is worth noting that pan-cytokeratin IHC can be misleading in the context of pseudobudding, since individual keratin positive cells can be mistaken for TB when viewed without morphologic context. Therefore, keratin stains are probably best avoided when the H&E features suggest pseudobudding. A recent study suggests that cancer gland rupture may be linked to LNM in pT1 CRC [15], but this remains to be confirmed by other groups. Until the relationship between gland rupture, TB, and LNM is better understood, pseudobudding should be excluded from the TB assessment. Finally, heavy inflammatory infiltrates may sometimes obscure TB, while reactive inflammatory and stromal cells can be difficult to distinguish from TB. In this setting, pan-cytokeratin IHC can be very helpful to better visualize TB, although the final bud count should be performed on H&E. Most participants indicated that they use pan-cytokeratin IHC in this scenario to aid visualization of TB.

The role of IHC alone in TB scoring remains controversial, with most participants indicating that more evidence is required before this can be considered routine practice. There was consensus that criteria for TB scoring based on IHC would need to differ from those based on H&E [16] since higher thresholds must be reached before TB assessed by H&E assumes prognostic significance [17, 18]. In addition, risk stratification does not appear to be improved with the use of IHC [16]. Some studies have shown improved reproducibility in TB scoring with IHC compared to H&E [19, 20], while others have not [21, 22]. While pan-cytokeratin IHC increases the sensitivity of TB detection, it is also associated with its own unique challenges. In particular, the nuclei of tumor buds are not always clearly visualized on IHC. This can be problematic if the presence of a nucleus is used as a minimum criterion for a tumor bud, as proposed in some studies [16]. Moreover, pseudobudding produced by inflammation-induced fragmentation, mechanical causes, or treatment may be difficult to distinguish from true TB on IHC stains alone since these may not capture the morphologic context. These challenges might explain the only moderate interobserver agreement reported among expert gastrointestinal pathologists at the individual tumor bud level, which was no better for pan-cytokeratin than for H&E [16]. Overall, most studies have shown TB scoring assessed with either IHC or H&E to be in the moderate or substantial range [21, 23–26], although this may be lower among non-subspecialist GI pathologists [27].

With regard to locally resected pT1 cancers, there was strong consensus that TB scoring should be routinely performed in practice, incorporated in national guidelines, and be factored into clinical decision-making. This is supported by strong evidence establishing intermediate and high TB (Bd2/Bd3) as independent predictors of LNM in pT1 CRC [28–30]. There was also consensus that locally resected pT1 cancers, in which intermediate or high TB was the only high-risk feature, should be discussed in a multidisciplinary meeting. In such cases, the decision regarding the need for surgical resection should take into account clinical factors, including operative risk and comorbidities, in order to balance the risks of over- and under-treatment. In pT1 cancers without TB (Bd1) or any other adverse risk factors, the risk of LNM is very low, and endoscopic resection is generally considered sufficient [31].

With regard to stage II CRC, there was consensus that TB scoring should be performed in daily practice, that TB should be included in national guidelines, and that clinicians be aware of its presence. There was also consensus that CRC with high TB (Bd3) should be considered at high risk for subsequent recurrence. This reflects strong evidence that high TB (Bd3) is an independent predictor of recurrence and mortality in stage II CRC [24, 32–36]. Consensus was not reached on whether clinical decisions should be made on the basis of TB alone in this setting. Adjuvant chemotherapy is currently not recommended for stage II CRC without high-risk features since the absolute benefits have been shown to be very small [37]. However, most oncology guidelines recommend that adjuvant chemotherapy be considered with high-risk stage II CRC [38]. Data from QUASAR [37] and SACURA [12] trials confirm the adverse prognostic value of TB in large cohorts of stage II CRC and suggest adjuvant chemotherapy may be beneficial in such patients. However, further prospective clinical trials are needed to confirm the benefit of adjuvant chemotherapy in stage II CRC with high TB.

The significance of the Bd2 category in the 3-tiered ITBCC scoring system is a potential source of confusion and may require some clarification. The Bd2 category assumes differing prognostic significance depending on the clinical scenario (i.e., pT1 or stage II CRC). Given the strong evidence that 5 or more tumor buds in a 0.785mm^2^ field is an independent predictor of LNM in pT1 CRC, Bd2 is considered a “high risk” in this setting. In contrast, in stage II CRC, the most significant risk for recurrence and mortality is seen when TB counts reach 10 or more (i.e., Bd3). As such, the Bd2 category is not considered a “high-risk” category in stage II CRC. Most participants (64%) agreed with the statement that “Bd2 can be considered either part of low TB (Stage II CRC) or high TB (pT1 CRC),” while 29% expressed “no opinion” and 7% disagreed. The lack of consensus on this statement may reflect the fact that Bd2, while not “high risk” in stage II, is not strictly “low risk” either, since the risk is intermediate between Bd1 and Bd3. However, for practical purposes, in pT1 CRC, Bd2 should be considered a risk factor for LNM, while in stage II CRC, Bd2 is not considered a high-risk feature.

TB shares several features in common with poorly differentiated clusters (PDC) from which they are distinguished by an arbitrary numerical cut-off (PDCs are defined as clusters of 5 or more tumor cells lacking glandular structure). PDCs have gained increasing recognition as an invasive front prognostic marker in CRC [39]. It has been suggested that PDC and TB may be part of a biologic continuum and reflect different stages of cancer cell invasion (#26). Although some studies have shown PDCs and TB to share biological similarities [40, 41], their relationship requires further investigation. There was consensus that, until more evidence regarding their underlying biology is available, TB and PDCs are best considered different and evaluated separately. It was acknowledged, however, that TB may arise from PDCs in addition to the main tumor mass.

Automated detection algorithms for TB, applied to either H&E- or IHC-stained sections, could prove effective in advancing our knowledge of TB and incorporating TB into routine clinical practice. Examples of these algorithms for TB in IHC have begun to emerge [42], yet there is still a need for a reliable algorithm that can automatically detect TB on H&E. There are several ways in which the digital interpretation of TB could help pathologists, such as detecting TB across an entire tissue slide, identifying hotspot areas, and potentially providing a TB score automatically. This will not come without its share of challenges but may serve to improve the efficiency, accuracy, and reproducibility of TB scoring which are all barriers to widespread implementation currently. In the research setting, digital interpretation of TB can help provide insight into how peritumoral budding, which is TB at the invasive margin, compares with intratumoral budding, which is TB within the tumor bulk [43]. How TB compares to PDCs prognostically, how current scoring cut-offs are set, and more dynamic scoring systems, such as continuous scaling method or scoring within multiple hotspots, can also be explored.

Lastly, a number of unresolved issues related to TB have been identified which require further research (Box 2). In conclusion, the standardized assessment and scoring system for TB established by the ITBCC 2016 have been incorporated into a number of international CRC guidelines and validated in large cohorts of CRC patients. We used a modified Delphi survey and in-person meeting to evaluate new evidence, generate consensus on a number of issues related to TB in CRC, and highlight areas in need of further research. This process has re-affirmed the importance of TB in CRC and supports its continued use in routine clinical practice. New technologies such as automated detection algorithms will be critical to improving the way TB assessment is conducted and implemented in clinical practice.
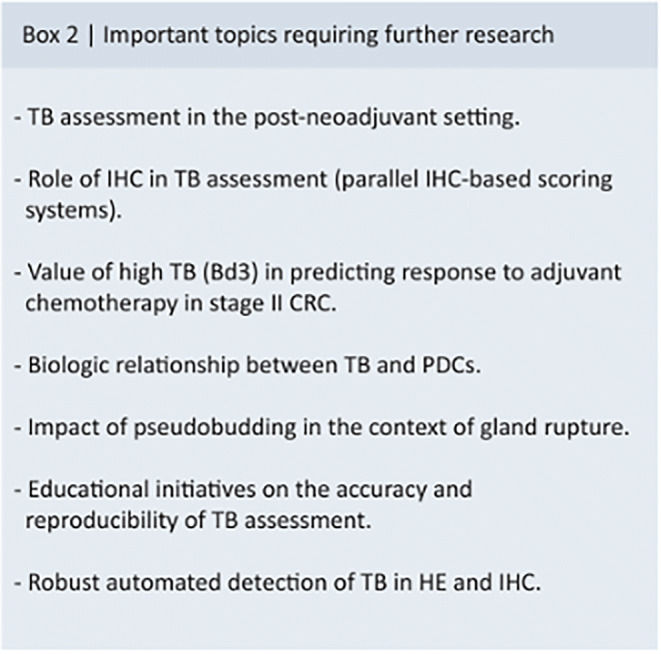


## Supplementary Information

ESM 1 (DOCX 22 kb)
