# Supplementary material for: Improving tumor budding reporting in colorectal cancer: a Delphi consensus study
Source: Virchows Arch. 2021 Mar 1;479(3):459–69. doi: 10.1007/s00428-021-03059-9 (PMC8448718; doi:10.1007/s00428-021-03059-9)
Supplement: Supplementary file 1 — (DOCX 22 kb) [file 428_2021_3059_MOESM1_ESM.docx]

**Supplementary Material**

**Supplementary Data Table 1**

Statements not achieving consensus in Round 1.

| **Round 1** |
| --- |
| The tumor budding definition should be modified or adapted by digital pathology. |
| The tumor budding should be modified or adapted by immunohistochemistry. |
| Tumor budding should be used in all colorectal cancer irrespective of its stage. |
| Stage II – My clinicians should make decisions based on tumor budding. |

**Supplementary Data Table 2**

Statements not achieving consensus in Round 2.

| **Round 2** |
| --- |
| In case of a BD3 CRC, where this is the only risk factor, I would advise surgical resection based on BD alone. |
| In case of budding, I always apply a D2-40 stain to determine whether it is true budding or lymphatic invasion. |
| I use IHC (cytokeratin) in this setting, to determine the presence of tumor budding. |
| For budding based on IHC and budding on H&E, I use the same criteria. |
| I take budding into account, but only report this as a combination with other risk factors (risk stratification model). |
| Tumor budding is included in my national guidelines as a core item. |
| Tumor budding is included in my national guidelines as a non-core item. |
| In case of Bd3 as the only risk factor, I would consider this case high risk stage II and advise adjuvant chemotherapy. |
| Budding and PDCs have similar prognostic value. |
| Budding and PDCs form a morphologic continuum. |
| The definition of TB should be modified to incorporate PDCs. |
| Interobserver variability for TB is higher in pT1 CRC compared to CRC resection specimens of larger tumors. |
| Classification of TB should be dependent on the clinical scenario (i.e. there are differences between cases with local excision and surgical resection). |
| The definition of TB should be modified to incorporate IHC. |

**Supplementary Data Figure 1**

Literature reviewed to generate consensus statements and the contents of the e-book distributed to the study participants.

1. Almangush A, Youssef O, Pirinen M, Sundstrom J, Leivo I, Makitie AA (2019) Does evaluation of tumour budding in diagnostic biopsies have a clinical relevance? A systematic review Histopathology 74:536-544. doi: 10.1111/his.13793

2. Backes Y, Elias SG, Groen JN, Schwartz MP, Wolfhagen FHJ, Geesing JMJ, Ter Borg F, van Bergeijk J, Spanier BWM, de Vos Tot Nederveen Cappel WH, Kessels K, Seldenrijk CA, Raicu MG, Drillenburg P, Milne AN, Kerkhof M, Seerden TCJ, Siersema PD, Vleggaar FP, Offerhaus GJA, Lacle MM, Moons LMG, Dutch TCRCWG (2018) Histologic Factors Associated With Need for Surgery in Patients With Pedunculated T1 Colorectal Carcinomas Gastroenterology 154:1647-1659. doi: 10.1053/j.gastro.2018.01.023

3. Barel F, Auffret A, Cariou M, Kermarrec T, Samaison L, Bourhis A, Badic B, Jezequel J, Cholet F, Bail JP, Marcorelles P, Nousbaum JB, Robaszkiewicz M, Doucet L, Uguen A (2019) High reproducibility is attainable in assessing histoprognostic parameters of pT1 colorectal cancer using routine histopathology slides and immunohistochemistry analyses Pathology 51:46-54. doi: 10.1016/j.pathol.2018.10.007

4. Barel F, Cariou M, Saliou P, Kermarrec T, Auffret A, Samaison L, Bourhis A, Badic B, Jezequel J, Cholet F, Bail JP, Marcorelles P, Nousbaum JB, Robaszkiewicz M, Doucet L, Uguen A (2019) Histopathological factors help to predict lymph node metastases more efficiently than extra-nodal recurrences in submucosa invading pT1 colorectal cancer Sci Rep 9:8342. doi: 10.1038/s41598-019-44894-w

5. Beaton C, Twine CP, Williams GL, Radcliffe AG (2013) Systematic review and meta-analysis of histopathological factors influencing the risk of lymph node metastasis in early colorectal cancer Colorectal Dis 15:788-797. doi: 10.1111/codi.12129

6. Betge J, Kornprat P, Pollheimer MJ, Lindtner RA, Schlemmer A, Rehak P, Vieth M, Langner C (2012) Tumor budding is an independent predictor of outcome in AJCC/UICC stage II colorectal cancer Ann Surg Oncol 19:3706-3712. doi: 10.1245/s10434-012-2426-z

7. Reggiani Bonetti L, Barresi V, Maiorana A, Manfredini S, Caprera C, Bettelli S (2018) Clinical Impact and Prognostic Role of KRAS/BRAF/PIK3CA Mutations in Stage I Colorectal Cancer Dis Markers 2018:2959801. doi: 10.1155/2018/2959801

8. Bosch SL, Teerenstra S, de Wilt JH, Cunningham C, Nagtegaal ID (2013) Predicting lymph node metastasis in pT1 colorectal cancer: a systematic review of risk factors providing rationale for therapy decisions Endoscopy 45:827-834. doi: 10.1055/s-0033-1344238

9. Cappellesso R, Luchini C, Veronese N, Lo Mele M, Rosa-Rizzotto E, Guido E, De Lazzari F, Pilati P, Farinati F, Realdon S, Solmi M, Fassan M, Rugge M (2017) Tumor budding as a risk factor for nodal metastasis in pT1 colorectal cancers: a meta-analysis Hum Pathol 65:62-70. doi: 10.1016/j.humpath.2017.04.013

10. Cho S-J, Kakar S (2018) Tumor Budding in Colorectal Carcinoma Arch Pathol Lab Med 142:952-957. doi: 10.5858/

11. Dawson H, Galuppini F, Trager P, Berger MD, Studer P, Brugger L, Zlobec I, Inderbitzin D, Lugli A (2019) Validation of the International Tumor Budding Consensus Conference 2016 recommendations on tumor budding in stage I-IV colorectal cancer Hum Pathol 85:145-151. doi: 10.1016/j.humpath.2018.10.023

12. De Smedt L, Palmans S, Andel D, Govaere O, Boeckx B, Smeets D, Galle E, Wouters J, Barras D, Suffiotti M, Dekervel J, Tousseyn T, De Hertogh G, Prenen H, Tejpar S, Lambrechts D, Sagaert X (2017) Expression profiling of budding cells in colorectal cancer reveals an EMT-like phenotype and molecular subtype switching Br J Cancer 116:58-65. doi: 10.1038/bjc.2016.382

13. Demir A, Alan O, Oruc E (2019) Tumor budding for predicting prognosis of resected rectum cancer after neoadjuvant treatment World J Surg Oncol 17:50. doi: 10.1186/s12957-019-1588-6

14. Enderle-Ammour K, Wellner U, Kocsmar E, Kiss A, Lotz G, Csanadi A, Bader M, Schilling O, Werner M, Bronsert P (2018) [Three-dimensional reconstruction of solid tumors : Morphological evidence for tumor heterogeneity] Pathologe 39:231-235. doi: 10.1007/s00292-018-0529-4

15. Eriksen AC, Andersen JB, Lindebjerg J, dePont Christensen R, Hansen TF, Kjaer-Frifeldt S, Sorensen FB (2018) Does heterogeneity matter in the estimation of tumour budding and tumour stroma ratio in colon cancer? Diagn Pathol 13:20. doi: 10.1186/s13000-018-0697-9

16. Eriksen AC, Sorensen FB, Lindebjerg J, Hager H, dePont Christensen R, Kjaer-Frifeldt S, Hansen TF (2018) The prognostic value of tumour stroma ratio and tumour budding in stage II colon cancer. A nationwide population-based study Int J Colorectal Dis 33:1115-1124. doi: 10.1007/s00384-018-3076-9

17. Graham RP, Vierkant RA, Tillmans LS, Wang AH, Laird PW, Weisenberger DJ, Lynch CF, French AJ, Slager SL, Raissian Y, Garcia JJ, Kerr SE, Lee HE, Thibodeau SN, Cerhan JR, Limburg PJ, Smyrk TC (2015) Tumor Budding in Colorectal Carcinoma: Confirmation of Prognostic Significance and Histologic Cutoff in a Population-based Cohort Am J Surg Pathol 39:1340-1346. doi: 10.1097/PAS.0000000000000504

18. Grigore AD, Jolly MK, Jia D, Farach-Carson MC, Levine H (2016) Tumor Budding: The Name is EMT. Partial EMT J Clin Med 5. doi: 10.3390/jcm5050051

19. Hashimoto H, Horiuchi H, Kurata A, Kikuchi H, Okuyama R, Usui G, Masuda Y, Kuroda M, Inoue S, Furushima K, Matsuhashi N, Harihara Y, Morikawa T (2019) Intramucosal colorectal carcinoma with lymphovascular invasion: clinicopathological characteristics of nine cases Histopathology 74:1055-1066. doi: 10.1111/his.13826

20. Huh JW, Lee WY, Shin JK, Park YA, Cho YB, Kim HC, Yun SH (2019) A novel histologic grading system based on lymphovascular invasion, perineural invasion, and tumor budding in colorectal cancer Journal of Cancer Research and Clinical Oncology 145:471-477. doi: 10.1007/s00432-018-2804-4

21. Jager T, Neureiter D, Fallaha M, Schredl P, Kiesslich T, Urbas R, Klieser E, Holzinger J, Sedlmayer F, Emmanuel K, Dinnewitzer A (2018) The potential predictive value of tumor budding for neoadjuvant chemoradiotherapy response in locally advanced rectal cancer Strahlenther Onkol 194:991-1006. doi: 10.1007/s00066-018-1340-0

22. Jass JR, Barker M, Fraser L, Walsh MD, Whitehall VL, Gabrielli B, Young J, Leggett BA (2003) APC mutation and tumour budding in colorectal cancer J Clin Pathol 56:69-73

23. Jepsen RK, Klarskov LL, Lippert MF, Novotny GW, Hansen TP, Christensen IJ, Hogdall E, Riis LB (2018) Digital image analysis of pan-cytokeratin stained tumor slides for evaluation of tumor budding in pT1/pT2 colorectal cancer: Results of a feasibility study Pathol Res Pract 214:1273-1281. doi: 10.1016/j.prp.2018.07.002

24. Kakar S, Chanjuan S, Mariana BE, Driman DK, Fitzgibbons P, Frankel W, Hill KA, Jessup J, Krasinskas AM, Washington MK (2017) Protocol for the examination of specimens from patients with primary carcinoma of the colon and rectum. <https://documents.cap.org/protocols/cp-gilower-colonrectum-17protocol-4010.pdf>

25. Karlberg M, Stenstedt K, Hallstrom M, Ragnhammar P, Lenander C, Edler D (2018) Tumor Budding Versus Mismatch Repair Status in Colorectal Cancer - An Exploratory Analysis Anticancer Res 38:4713-4721. doi: 10.21873/anticanres.12778

26. Knudsen KN, Lindebjerg J, Kalmar A, Molnar B, Sorensen FB, Hansen TF, Nielsen BS (2018) miR-21 expression analysis in budding colon cancer cells by confocal slide scanning microscopy Clin Exp Metastasis 35:819-830. doi: 10.1007/s10585-018-9945-3

27. Landau MA, Zhu B, Akwuole FN, Pai RK (2019) Histopathological Predictors of Recurrence in Stage III Colon Cancer: Reappraisal of Tumor Deposits and Tumor Budding Using AJCC8 Criteria Int J Surg Pathol 27:147-158. doi: 10.1177/1066896918787275

28. Lang-Schwarz C, Melcher B, Haumaier F, Schneider-Fuchs A, Lang-Schwarz K, Krugmann J, Vieth M, Sterlacci W (2019) Budding, tumor-infiltrating lymphocytes, gland formation: scoring leads to new prognostic groups in World Health Organization low-grade colorectal cancer with impact on survival Hum Pathol 89:81-89. doi: 10.1016/j.humpath.2019.04.006

29. Lee VWK, Chan KF (2018) Tumor budding and poorly-differentiated cluster in prognostication in Stage II colon cancer Pathol Res Pract 214:402-407. doi: 10.1016/j.prp.2017.12.019

30. Lino-Silva LS, Salcedo-Hernandez RA, Gamboa-Dominguez A (2018) Tumour budding in rectal cancer. A comprehensive review Contemp Oncol (Pozn) 22:61-74. doi: 10.5114/wo.2018.77043

31. Lino-Silva LS, Gamboa-Domínguez A, Zúñiga-Tamayo D, Salcedo-Hernández RA, Cetina L, Cantú-de-León D (2018) Mismatch repair protein expression and intratumoral budding in rectal cancer are associated with an increased pathological complete response to preoperative chemoradiotherapy: A case-control study World Journal of Clinical Oncology 9:133-139. doi: 10.5306/wjco.v9.i7.133

32. Lugli A, Kirsch R, Ajioka Y, Bosman F, Cathomas G, Dawson H, El Zimaity H, Flejou JF, Hansen TP, Hartmann A, Kakar S, Langner C, Nagtegaal I, Puppa G, Riddell R, Ristimaki A, Sheahan K, Smyrk T, Sugihara K, Terris B, Ueno H, Vieth M, Zlobec I, Quirke P (2017) Recommendations for reporting tumor budding in colorectal cancer based on the International Tumor Budding Consensus Conference (ITBCC) 2016 Mod Pathol 30:1299-1311. doi: 10.1038/modpathol.2017.46

33. Martin B, Schafer E, Jakubowicz E, Mayr P, Ihringer R, Anthuber M, Schenkirsch G, Schaller T, Markl B (2018) Interobserver variability in the H&E-based assessment of tumor budding in pT3/4 colon cancer: does it affect the prognostic relevance? Virchows Arch 473:189-197. doi: 10.1007/s00428-018-2341-1

34. Martinez-Ciarpaglini C, Oltra S, Rosello S, Roda D, Mongort C, Carrasco F, Gonzalez J, Santonja F, Tarazona N, Huerta M, Espi A, Ribas G, Ferrandez A, Navarro S, Cervantes A (2019) Low miR200c expression in tumor budding of invasive front predicts worse survival in patients with localized colon cancer and is related to PD-L1 overexpression Mod Pathol 32:306-313. doi: 10.1038/s41379-018-0124-5

35. Mehta A, Goswami M, Sinha R, Dogra A (2018) Histopathological Significance and Prognostic Impact of Tumor Budding in Colorectal Cancer Asian Pac J Cancer Prev 19:2447-2453. doi: 10.22034/APJCP.2018.19.9.2447

36. Moller T, James JP, Holmstrom K, Sorensen FB, Lindebjerg J, Nielsen BS (2019) Co-Detection of miR-21 and TNF-alpha mRNA in Budding Cancer Cells in Colorectal Cancer Int J Mol Sci 20. doi: 10.3390/ijms20081907

37. Oh JR, Park B, Lee S, Han KS, Youk E-G, Lee D-H, Kim D-S, Lee D-S, Hong CW, Kim BC, Kim B, Kim MJ, Park SC, Sohn DK, Chang HJ, Oh JH (2019) Nomogram Development and External Validation for Predicting the Risk of Lymph Node Metastasis in T1 Colorectal Cancer Cancer Research and Treatment 51:1275-1284. doi: 10.4143/crt.2018.569

38. Pai RK, Cheng YW, Jakubowski MA, Shadrach BL, Plesec TP, Pai RK (2017) Colorectal carcinomas with submucosal invasion (pT1): analysis of histopathological and molecular factors predicting lymph node metastasis Mod Pathol 30:113-122. doi: 10.1038/modpathol.2016.166

39. Lee SJ, Kim A, Kim YK, Park WY, Kim HS, Jo HJ, Oh N, Song GA, Park DY (2018) The significance of tumor budding in T1 colorectal carcinoma: the most reliable predictor of lymph node metastasis especially in endoscopically resected T1 colorectal carcinoma Hum Pathol 78:8-17. doi: 10.1016/j.humpath.2018.02.001

40. Risio M (2012) The Natural History of pT1 Colorectal Cancer Front Oncol 2:22. doi: 10.3389/fonc.2012.00022

41. Risio M (2012) The natural history of colorectal adenomas and early cancer Pathologe 33 Suppl 2:206-210. doi: 10.1007/s00292-012-1640-6

42. Rogers AC, Winter DC, Heeney A, Gibbons D, Lugli A, Puppa G, Sheahan K (2016) Systematic review and meta-analysis of the impact of tumour budding in colorectal cancer Br J Cancer 115:831-840. doi: 10.1038/bjc.2016.274

43. Romiti A, Roberto M, Marchetti P, Di Cerbo A, Falcone R, Campisi G, Ferri M, Balducci G, Ramacciato G, Ruco L, Pilozzi E (2019) Study of histopathologic parameters to define the prognosis of stage II colon cancer Int J Colorectal Dis 34:905-913. doi: 10.1007/s00384-019-03279-1

44. Roseweir AK, Kong CY, Park JH, Bennett L, Powell A, Quinn J, van Wyk HC, Horgan PG, McMillan DC, Edwards J, Roxburgh CS (2018) A novel tumor-based epithelial-to-mesenchymal transition score that associates with prognosis and metastasis in patients with stage II/III colorectal cancer Int J Cancer. doi: 10.1002/ijc.31739

45. Oh BY, Park YA, Huh JW, Yun SH, Kim HC, Chun H-K, Kim SH, Ha SY, Lee WY, Cho YB (2017) Prognostic Impact of Tumor-Budding Grade in Stages 1–3 Colon Cancer: A Retrospective Cohort Study Annals of Surgical Oncology 25:204-211. doi: 10.1245/s10434-017-6135-5

46. De Smedt L, Palmans S, Sagaert X (2016) Tumour budding in colorectal cancer: what do we know and what can we do? Virchows Arch 468:397-408. doi: 10.1007/s00428-015-1886-5

47. Sirin AH, Sokmen S, Unlu SM, Ellidokuz H, Sarioglu S (2019) The prognostic value of tumor budding in patients who had surgery for rectal cancer with and without neoadjuvant therapy Tech Coloproctol 23:333-342. doi: 10.1007/s10151-019-01959-2

48. Slik K, Blom S, Turkki R, Valimaki K, Kurki S, Mustonen H, Haglund C, Carpen O, Kallioniemi O, Korkeila E, Sundstrom J, Pellinen T (2019) Combined epithelial marker analysis of tumour budding in stage II colorectal cancer J Pathol Clin Res 5:63-78. doi: 10.1002/cjp2.119

49. Takamatsu M, Kawachi H, Yamamoto N, Kobayashi M, Toyama Y, Maekawa T, Chino A, Saito S, Ueno M, Takazawa Y, Ishikawa Y (2019) Immunohistochemical evaluation of tumor budding for stratifying T1 colorectal cancer: optimal cut-off value and a novel computer-assisted semiautomatic method Mod Pathol 32:675-683. doi: 10.1038/s41379-018-0189-1

50. Konishi T, Shimada Y, Lee LH, Cavalcanti MS, Hsu M, Smith JJ, Nash GM, Temple LK, Guillem JG, Paty PB, Garcia-Aguilar J, Vakiani E, Gonen M, Shia J, Weiser MR (2018) Poorly Differentiated Clusters Predict Colon Cancer Recurrence: An In-Depth Comparative Analysis of Invasive-Front Prognostic Markers Am J Surg Pathol 42:705-714. doi: 10.1097/PAS.0000000000001059

51. Ueno H, Mochizuki H, Hashiguchi Y, Shimazaki H, Aida S, Hase K, Matsukuma S, Kanai T, Kurihara H, Ozawa K, Yoshimura K, Bekku S (2004) Risk factors for an adverse outcome in early invasive colorectal carcinoma Gastroenterology 127:385-394. doi: 10.1053/j.gastro.2004.04.022

52. Ueno H, Murphy J, Jass JR, Mochizuki H, Talbot IC (2002) Tumor 'budding'as index to estimate potential aggressivenes in rectal cancer Histopathology 40:127-132

53. Ueno H, Ishiguro M, Nakatani E, Ishikawa T, Uetake H, Matsuda C, Nakamoto Y, Kotake M, Kurachi K, Egawa T, Yasumasa K, Murata K, Ikawa O (2019) Prospective Multicenter Study on the Prognostic and Predictive Impact of Tumor Budding in Stage II Colon Cancer: Results from the SACURA Trial Journal of Clinical Oncology 37:1886-1894. doi: 10.1200/JCO.18

54. Weis CA, Kather JN, Melchers S, Al-Ahmdi H, Pollheimer MJ, Langner C, Gaiser T (2018) Automatic evaluation of tumor budding in immunohistochemically stained colorectal carcinomas and correlation to clinical outcome Diagn Pathol 13:64. doi: 10.1186/s13000-018-0739-3

55. Yamadera M, Shinto E, Kajiwara Y, Mochizuki S, Okamoto K, Shimazaki H, Hase K, Ueno H (2019) Differential clinical impacts of tumour budding evaluated by the use of immunohistochemical and haematoxylin and eosin staining in stage II colorectal cancer Histopathology 74:1005-1013. doi: 10.1111/his.13830

56. Zhang Q, Wang L, Huang D, Xu M, Weng W, Ni S, Tan C, Sheng W (2019) Pathological risk factors for lymph node metastasis in patients with submucosal invasive colorectal carcinoma Cancer Manag Res 11:1107-1114. doi: 10.2147/CMAR.S181740

57. Trinh A, Ladrach C, Dawson HE, Ten Hoorn S, Kuppen PJK, Reimers MS, Koopman M, Punt CJA, Lugli A, Vermeulen L, Zlobec I (2018) Tumour budding is associated with the mesenchymal colon cancer subtype and RAS/RAF mutations: a study of 1320 colorectal cancers with Consensus Molecular Subgroup (CMS) data Br J Cancer 119:1244-1251. doi: 10.1038/s41416-018-0230-7

58. Zlobec I, Bihl MP, Foerster A, Rufle A, Lugli A (2012) The impact of CpG island methylator phenotype and microsatellite instability on tumour budding in colorectal cancer Histopathology 61:777-787. doi: 10.1111/j.1365-2559.2012.04273.x
